# Colorectal Cancer Screening

**DOI:** 10.1155/2012/476065

**Published:** 2012-08-16

**Authors:** Enrique Quintero, Yutaka Saito, Cessare Hassan, Carlo Senore

**Affiliations:** ^1^Department of Gastroenterology, University Hospital of the Canary Islands, La Laguna University School of Medicine, 38320 Tenerife, Spain; ^2^Gastrointestinal Endoscopy Division, National Cancer Center Hospital, 104-0045 Tokyo, Japan; ^3^Department of Gastroenterology, Nuovo Regina Margherita Hospital, 00153 Rome, Italy; ^4^SCDO Epidemiologia dei Tumori, Azienda Ospedaliero-Universitaria San Giovanni Battista, 10126 Torino, Italy

Colorectal cancer (CRC) is the most common malignancy worldwide if men and women are considered together, with more than 1,200,000 new cases per year. The highest incidence rates are found in Australia and New Zealand, Europe, and North America. In addition, it represents, after lung cancer, the second leading cause of cancer-related death among these populations.

CRC can be preventable as more than 85% of tumors arise in a premalignant polyp. Therefore, the aim of CRC screening is to reduce mortality by identifying individuals with pre-symptomatic neoplastic lesions who may require further examination and treatment. Screening tests should be workable, unexpensive, acceptable, sensitive, specific, and safe. However, none of the available recommended tests for CRC screening fulfill these premises. At present, the most acceptable strategies for screening in the average-risk population (individuals without a family history of CRC between 50 and 74 years) are fecal occult blood test (FOBT) every year or biannually, sigmoidoscopy every 5 years, and colonoscopy every 10 years. Other provocative procedures such us computed tomography colonography, faecal DNA analysis, and other molecular tests have emerged as an alternative method during the last decade and are actually under evaluation. By contrast, in individuals with familial risk (first-degree relatives of patients with CRC), colonoscopy is empirically recommended from age 40 or 10 years before the age of diagnosis of the youngest relative.

Compliance is one of the main factors influencing the success of any population-based screening program. If the test is too complicated to perform or not easily accepted by the target population, the participation rate will be poor and the effectiveness of the program will be low. In fact, this is a relevant drawback in many CRC screening programs running worldwide, with compliance rates between 25% and 67%.

In the current special issue, worldwide experts address several controversial aspects related to CRC screening in average-risk population. There are two papers analyzing the main barriers and interventions that may determine screening uptake. A. Z. Gimeno García reviews the factors related to CRC screening behavior and the interventions that may improve screening uptake, whereas G. G. Medina et al. are reporting the results of a survey that explores the main barriers experienced by screeners and nonscreeners. In addition, the effectiveness of molecular fecal testing and the applicability of image-enhanced endoscopy to improve early detection of colorectal neoplasms is approached in three additional papers. R. Kanthan et al. analyze the genetic and epigenetic fecal molecular markers that are being implemented for the identification of malignant and premalignant colorectal lesions; H. Aihara and H. Tajiri reviewed the advantages and limitations of autofluorescence endoscopy for CRC screening, suggesting this technique as a potential tool for improving CRC-related mortality in the forthcoming years; N. Tamai and coworkers actually show the benefit of autofluorescence imaging over narrow-binding image, chromoendoscopy with indigo carmine, and white-light endoscopy, for detecting laterally spreading colorectal tumors. Moreover, M. Sekiguchi et al. compare cost effectiveness of one-time colonoscopy screening versus biennial fecal immunochemical testing in a retrospective study carried out in Japan. They conclude that colonoscopy is a cost-effective screening strategy in the Japanese average-risk population. Finally, C. Senore, C. Hassan, Y. Saito, and E. Quintero summarize the recent advances regarding strategies for CRC screening, they address the novel implemented endoscopic technologies to improve the detection of nonprotruding neoplasms, and finally they discuss the factors influencing cost effectiveness in the setting of CRC screening.

*Enrique Quintero* *Enrique Quintero* *Yutaka Saito* *Yutaka Saito* *Cessare Hassan* *Cessare Hassan* *Carlo Senore* *Carlo Senore*

